# Human Leukocyte Antigen G Polymorphism and Expression Are Associated with an Increased Risk of Non-Small-Cell Lung Cancer and Advanced Disease Stage

**DOI:** 10.1371/journal.pone.0161210

**Published:** 2016-08-12

**Authors:** Amira Ben Amor, Karine Beauchemin, Marie-Claude Faucher, Agnes Hamzaoui, Kamel Hamzaoui, Michel Roger

**Affiliations:** 1 Tunis El Manar University, Medicine Faculty of Tunis, Department of Immunology and Histology, Tunis, Tunisia; 2 Laboratoire d’immunogénétique, Centre de Recherche du Centre Hospitalier de l’Université de Montréal (CRCHUM), Montreal, Canada; 3 Abderrahmen Mami Hospital, Unit Research UR/12SP15 (Homeostasis and cell immune dysfunction), Ariana, Tunisia; 4 Département de Microbiologie et Immunologie de l‘Université de Montréal, Montreal, Canada; Istituto di Ricovero e Cura a Carattere Scientifico Centro di Riferimento Oncologico della Basilicata, ITALY

## Abstract

Human leukocyte antigen (HLA)-G acts as negative regulator of the immune responses and its expression may enable tumor cells to escape immunosurveillance. The purpose of this study was to investigate the influence of *HLA-G* allelic variants and serum soluble HLA-G (sHLA-G) levels on risk of non-small-cell lung cancer (NSCLC). We analyzed 191 Caucasian adults with NSCLC and 191 healthy subjects recruited between January 2009 and March 2014 in Ariana (Tunisia). Serum sHLA-G levels were measured by immunoassay and *HLA-G* alleles were determined using a direct DNA sequencing procedures. The heterozygous genotypes of *HLA-G 010101* and -*G 010401* were associated with increased risks of both NSCLC and advanced disease stages. In contrast, the heterozygous genotypes of *HLA-G 0105N* and -*G 0106* were associated with decreased risks of NSCC and clinical disease stage IV, respectively. Serum sHLA-G levels were significantly higher in patients with NSCLC and particularly in those with advanced disease stages compared to healthy subjects. The area under the receiver-operating characteristic (ROC) curves was 0.82 for controls vs patients. Given 100% specificity, the highest sensitivity achieved to detect NSCLC was 52.8% at a cutoff value of 24.9 U/ml. Patients with the sHLA-G above median level (≥ 50 U/ml) had a significantly shorter survival time. This study demonstrates that *HLA-G* allelic variants are independent risk factors for NSCLC. Serum sHLA-G levels in NSCLC patients could be useful biomarkers for the diagnostic and prognosis of NSCLC.

## Introduction

Lung cancer is the most common malignancy and the leading cause of cancer mortality in the world [1,2]. The poor prognosis of lung cancer is largely due to the lack of symptoms until advanced stages, and screening tools that allow detection of early-stage tumors [3]. Thus, the investigation of appropriate tumor biomarkers for early detection is necessary for improved prognosis and a better survival rate.

The host immune system plays a crucial role in the detection and elimination of cancer cells by a process called the cancer immune surveillance [4]. During immune editing, tumor cells have developed different mechanisms to escape from this elimination [5,6]. HLA-G is a non-classical HLA class I molecule whose aberrant expression in a wide variety of cancers has been suggested as a mechanism by which tumour cells can escape immunosurveillance [7]. HLA-G acts as negative regulator of the immune response through several mechanisms including preventing antigen recognition and T-cell migration, and suppression of T and natural killer (NK) cells cytotoxic effects [7,8]. Aberrant HLA-G expression has been observed in lung cancer tissue [9–11] and relatively high blood soluble HLA-G (sHLA-G) levels have been associated with lung cancer and advanced clinical disease stages [12–14].

HLA-G exhibits low polymorphism with only 51 alleles described so far and HLA-G expression can be modulated by allelic variants [8]. *HLA-G* alleles have been reported to be associated with the risk of various types of tumors, including hepatocellular, esophageal, bladder, uterine cervical, childhood neuroblastoma and breast cancers [15–19]. However, the link between *HLA-G* alleles and lung cancer has not yet been studied. It remains to be elucidated whether the expression of HLA-G in the context of lung cancer depends on the grade/type of tumor or genetic background of patients or both.

## Materials and Methods

### The study population

A total of 191 Caucasian adults admitted to Abderrahmen Mami Hospital (Ariana, Tunisia) and diagnosed with non-small cell lung cancer (NSCLC) were collected between January 2009 and March 2014. Histological diagnosis and tumor stage were determined for all patients in accordance with the World Health Organization criteria and the TNM staging system of international union against cancer. The collection of samples was performed before patients begin their treatment. 191 controls subjects were selected from healthy blood volunteers’ donors, recruited from the National blood transfusion center of Tunisia at the same period. These controls were unrelated to patients with malignancies, and none had a personal history of malignancy at time of ascertainment. Written informed consent was obtained from each individual and the study was approved by the Institutional Ethics Committee of Abderrahmen Mami Hospital.

### Soluble HLA-G measurements and HLA-G genotyping

sHLA-G serum levels were measured using a sHLA-G Elisa kit (Exbio), which allows simultaneous detection of HLA-G1 and -G5 soluble proteins without discrimination. *HLA-G* alleles were determined by direct DNA sequencing analysis of the nucleotide regions encompassing exons 2–4 using purified DNA from blood samples as described previously [20].

### Statistical analysis

All analyses were performed using the Graph Pad PRISM 5.0 for Windows and Epi-Info statistical program 7.0. Participants were classified according to the disease status and descriptive statistics were used to describe pertinent variables. In order to assess the association between each of the *HLA-G* alleles with NSCLC or disease status, those subjects who were heterozygous and homozygous were compared separately with subjects who tested negatively for that allele. The association between each of the putative *HLA-G* alleles and risks of NSCLC and disease progression was investigated using adjusted multivariate logistic regression to derive odds ratios (ORs) and respective 95% confidence intervals (CI) as estimates of relative risks. Since control and patient groups were similar for age, gender and ethnicity, the models were adjusted only for smoking status. The analyses were restricted to those *HLA-G* alleles found at a frequency above 5% in the study population.

Overall patient survival was defined as the time from the date of diagnosis to the date of last follow-up (censored) or date of patient death by any cause (event). Survival probabilities were calculated using the Kaplan–Meier method. The comparison of the survival curves was carried out using the Log-rank test. The feasibility of using sHLA-G in serum as a potential biomarker for differentiating NSCLC patients from normal controls was assessed using the receiver-operating characteristic (ROC) curve analysis. P-values less than 0.05 were considered statistically significant in all analyses.

## Results

### Characteristic of the study population

The distribution of demographic and smoking status variables by disease status are outlined in Table 1. The study groups were similar with respect to age and gender. The average age of cases and controls was 58.7 and 54.1 years, respectively. The majority (89%) of both cases and controls were men. Smoking status was significantly different between groups. Most cases (90.6%) and less than half of controls (43.5%) were former or current smokers. Most of the cases (55.5%) had adenocarcinoma followed by squamous cell carcinoma (29.9%) and large cell carcinoma (14.6%), and 90% of patients had advanced disease stages (stages III and IV).

**Table 1:** Characteristics of cases and controls according to disease status.

Category	Controls	NSCL	Stage I-II	Stage III	Stage IV
N (%)	191	191	18 (9.4)	59 (30.9)	114 (59.7)
**Age**					
**Mean±SD (years)**	54.1 ± 6.7	58.7 ± 10.7	57.8 ± 11.8	61.3 ± 10.5	57.6 ± 10.3
**<50**	44 (23.0)	39 (20.4)	6 (33.3)	7 (11.86)	26 (22.8)
**≥50**	147 (77.0)	152 (79.6)	12 (66.7)	52 (88.13)	88 (77.2)
**Gender**					
**Male**	171 (89.5)	169 (88.5)	13 (72.2)	55 (93.2)	101 (88.6)
**Female**	20 (10.5)	22 (11.5)	5 (27.8)	4 (6.8)	13 (11.4)
**Smoking status**					
**Never**	108 (56.5)	18 (9.4)	2 (11.1)	3 (5.1)	13 (11.4)
**Former/current**	83 (43.5)	173 (90.6)	16 (88.9)	56 (94.9)	101 (88.6)

N: number; NSCLC: none small cell lung carcinoma; SD: standard deviation. P< 0.0001 between controls and all other categories for smoking status, P > 0.05 between controls and all other categories for age and gender calculated by Fisher’s exact test.

### Prevalence of HLA-G genotypes

Eleven *HLA-G* alleles were found in the study population. The wild-type HLA-G*01:*01*:*01* was the most prevalent allele (33.2%) among the controls, followed by G*01:*01*:*02* (21.2%), G*01:*03*:*01* (11.5%), G*01:*04*:*0*4 (10.7%), G*01:*06* (9.4%), G*01:*05N* (5.5%), G*01:*04*:*01* (3.9%), G*01:*01*:*03* (1.6%), G*01:*01*:*12* (1.6%), G*01:*01*:*14* (1.1%), and G*01:*01*:*17* (0.3%).

### HLA-G genotypes distribution and association with NSCLC and disease stages

Table 2 shows the distribution of the *HLA-G* genotypes by disease status while Table 3 shows the adjusted ORs and 95% CIs for the associations between *HLA-G* genotypes and NSCLC and disease stages for those with allele frequency of 5% or more. Some *HLA-G* genotypes were associated with altered risks of both NSCLC and advanced disease stages. The heterozygous genotypes of HLA-G*01:*01*:*01* and G*01:*04*:*01* were significantly associated with increased risks of both NSCLC (P = 0.029, P = 0.046, respectively) and advanced disease stages (P = 0.038, P = 0.030, respectively). In contrast, the heterozygous genotypes of HLA-G*01:*05N* and G*01:*06* were associated with a decreased risks of NSCC (P = 0.044) and clinical disease stage IV (P = 0.005), respectively. S1 and S2 Tables show the distribution and the adjusted ORs (95% CIs) for the associations between *HLA-G* genotypes and histological types, respectively. The heterozygous genotypes of HLA-G*01:*01*:*01* and G*01:*04*:*01* were associated with increased risks for all histopathological types reaching significant level for the adenocarcinoma lung cancer type which affected 55% of the patients of the present study. The heterozygous genotypes of HLA-G*01:*05N* and G*01:*06* were associated with decreased risks for all histological types but levels were not significant when analyzed separately. The lack of significant associations found when cases are dichotomized by the different histopathological types or clinical disease stages is presumably due to the relatively low number of patients per subgroups of histological types or disease clinical stages. This lack of sufficient power becomes more important with low prevalent alleles such as HLA-G*01:*05N* and G*01:*06*.

**Table 2:** Distribution of selected *HLA-G* genotypes by disease status.

Genotypes	Controls	NSCLC	Stage I-II	Stage III	Stage IV
N (%)	191	191	18 (9.4)	59 (30.9)	114 (59.7)
**010101**					
**Homozygote**	26 (13.6)	17 (8.9)	3 (16.7)	5 (8.5)	9 (7.9)
**Heterozygote**	75 (39.3)	110 (57.6)	9 (50.0	35 (59.3)	66 (57.9)
**Absent**	90 (47.1)	64 (33.5)	6 (33.3)	19 (32.2)	39 (34.2)
**010102**					
**Homozygote**	5 (2.6)	7 (3.7)	1 (5.6)	0 (0.0)	6 (5.3)
**Heterozygote**	71 (37.2)	46 (24.0)	3 (16.7)	15 (25.5)	28 (24.6)
**Absent**	115 (60.2)	138 (72.3)	14 (77.8)	44 (74.5)	80 (70.1)
**010301**					
**Homozygote**	5 (2.6)	1 (0.5)	0 (0.0)	1 (1.7)	0 (0.0)
**Heterozygote**	34 (17.8)	44 (23.1)	5 (27.8)	11 (18.6)	28 (24.6)
**Absent**	152 (79.6)	146 (76.4)	13 (72.2)	47 (79.7)	86 (75.4
**010401**					
**Homozygote**	0 (0.0)	1 (0.5)	0 (0.0)	0 (0.0)	1 (0.9)
**Heterozygote**	15 (7.9)	31 (16.2)	2 (11.1)	11 (18.6)	18 (15.8)
**Absent**	176 (92.1)	159 (83.3)	16 (88.9)	48 (81.4)	95 (83.3)
**010404**					
**Homozygote**	6 (3.1)	0 (0.0)	0 (0.0)	0 (0.0)	0 (0.0)
**Heterozygote**	29 (15.2)	24 (12.7)	2 (11.1)	4 (6.8)	18 (15.8)
**Absent**	156 (81.7)	167 (87.3)	16 (88.9)	55 (93.2)	96 (84.2)
**0105N**					
**Homozygote**	0 (0.0)	0 (0.0)	0 (0.0)	0 (0.0)	0 (0.0)
**Heterozygote**	21 (12.0)	12 (6.3)	0 (0.0)	3 (5.1)	9 (7.9)
**Absent**	170 (98.0)	179 (93.7)	18 (100)	56 (94.9)	105 (92.1)
**0106**					
**Homozygote**	0 (0.0)	14 (7.1)	2 (11.1)	5 (8.5)	7 (6.1)
**Heterozygote**	36 (18.8)	22 (11.7)	2 (11.1)	14 (23.7)	6 (5.3)
**Absent**	155 (81.2)	155 (81.2)	14 (77.8)	40 (67.8)	101 (88.6)

N: number, NSCLC: none small cell lung carcinoma. Selected *HLA-G* genotypes are those with a frequency above 5% in the study population

**Table 3:** Adjusted OR (CI) for the associations between selected *HLA-G* genotypes and lung cancer and disease stages.

Genotypes	NSCLC	Stage I-II	Stage III	Stage IV
**010101**				
**Homozygote**	0.84 [0.39–1.81]	1.68 [0.38–7.50]	0.81 [0.26–2.56]	0.74 [0.73–1.18]
**Heterozygote**	**1.74 [1.05–2.88]**	1.69 [0.55–5.21]	**2.04 [1.01–4.15]**	**1.81 [1.03–3.16]**
**Absent**	1.0	1.0	1.0	1.0
**010102**				
**Homozygote**	1.39 [0.35–5.60]	3.00 [0.29–38.8]	n.a	2.06 [0.50–8.54]
**Heterozygote**	0.61 [0.37–1.02]	0.41 [0.30–1.52]	0.62 [0.30–1.28]	0.65 [0.36–1.15]
**Absent**	1.0	1.0	1.0	1.0
**010301**				
**Homozygote**	n.a	n.a	0.48 [0.05–4.76]	n.a
**Heterozygote**	1.65 [0.90–3.0]	1.84 [0.58–5.87]	1.15 [0.15–2.70]	1.87 [0.97–3.60]
**Absent**	1.0	1.0	1.0	1.0
**010401**				
**Homozygote**	n.a	n.a	n.a	n.a
**Heterozygote**	**2.24 [1.04–4.81]**	1.52 [0.29–7.89]	**3.10 [1.11–8.52]**	2.14 [0.93–4.94]
**Absent**	1.0	1.0	1.0	1.0
**010404**				
**Homozygote**	n.a	n.a	n.a	n.a
**Heterozygote**	0.92 [0.46–1.88]	0.94 [0.19–4.68]	0.52 [0.16–1.72]	1.33 [0.74–2.78]
**Absent**	1.0	1.0	1.0	1.0
**0105N**				
**Homozygote**	n.a	n.a	n.a	n.a
**Heterozygote**	**0.43 [0.19–0.98]**	n.a	0.36 [0.10–1.34]	0.56 [0.23–1.36]
**Absent**	1.0	1.0	1.0	1.0
**0106**				
**Homozygote**	n.a	n.a	n.a	n.a
**Heterozygote**	0.57 [0.29–1.09]	0.59 [0.12–2.84]	2.38 [0.32–1.56]	**0.25 [0.10–0.66]**
**Absent**	1.0	1.0	1.0	1.0

CI, confidence interval; N.A: none applicable; NSCLC: none small cell lung carcinoma; OR, Odds ratio. Selected *HLA-G* genotypes are those with a frequency above 5% in the study population. Adjusted ORs for smoking status

### sHLA-G serum levels in the study population

**Fig 1:**
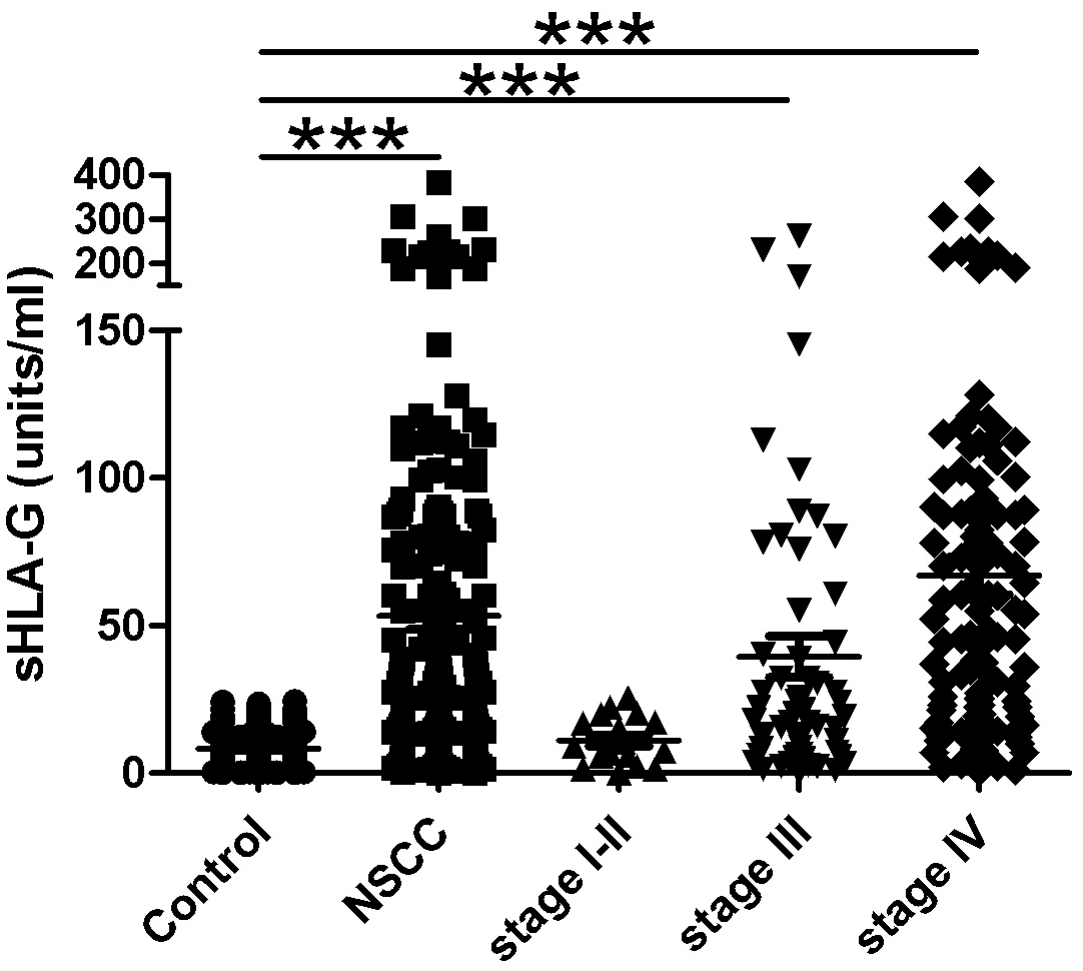
Serum sHLA-G levels in healthy controls and non-small-cell lung carcinoma (NSCLC) patients with different clinical stages. sHLA-G in all NSCLC patients and those with stages III and IV were significantly increased when compared to controls as determined by the Mann-Whitney U test (*** P< 0.0001).

The mean serum level of sHLA-G in NSCLC patients (53.3 ± 4.6 U/ml) was significantly increased compared to that found in controls (8.36 ± 0.4 U/ml), P< 0.0001 (Fig 1). The mean serum level of sHLA-G was increased independently of the histopathological type (S1 Fig). Furthermore mean serum sHLA-G levels in stages III (39.5 ± 7.0 U/ml) and IV (67 ± 6.5 U/ml) were higher than that observed in controls (8.36 ± 0.4 U/ml), P< 0.0001 (Fig 1). We also evaluated the correlation between serum sHLA-G levels and various characteristics of patients. No significant differences were found between males and females (51.4 ± 4.8 vs 67.2 ± 16 U/ml, P = 0.27) or between age groups of <50 and ≥50 years old (55.9 ± 11 vs 52.5 ± 5.1 U/ml; P = 0.76). Also no significant differences was found between none smoker and smoker groups (41.3 ± 13 vs 54.5 ± 4.9 U/ml; P = 0.40).

ROC curves were used to evaluate the performance of serum sHLA-G in detecting NSCLC.

**Fig 2:**
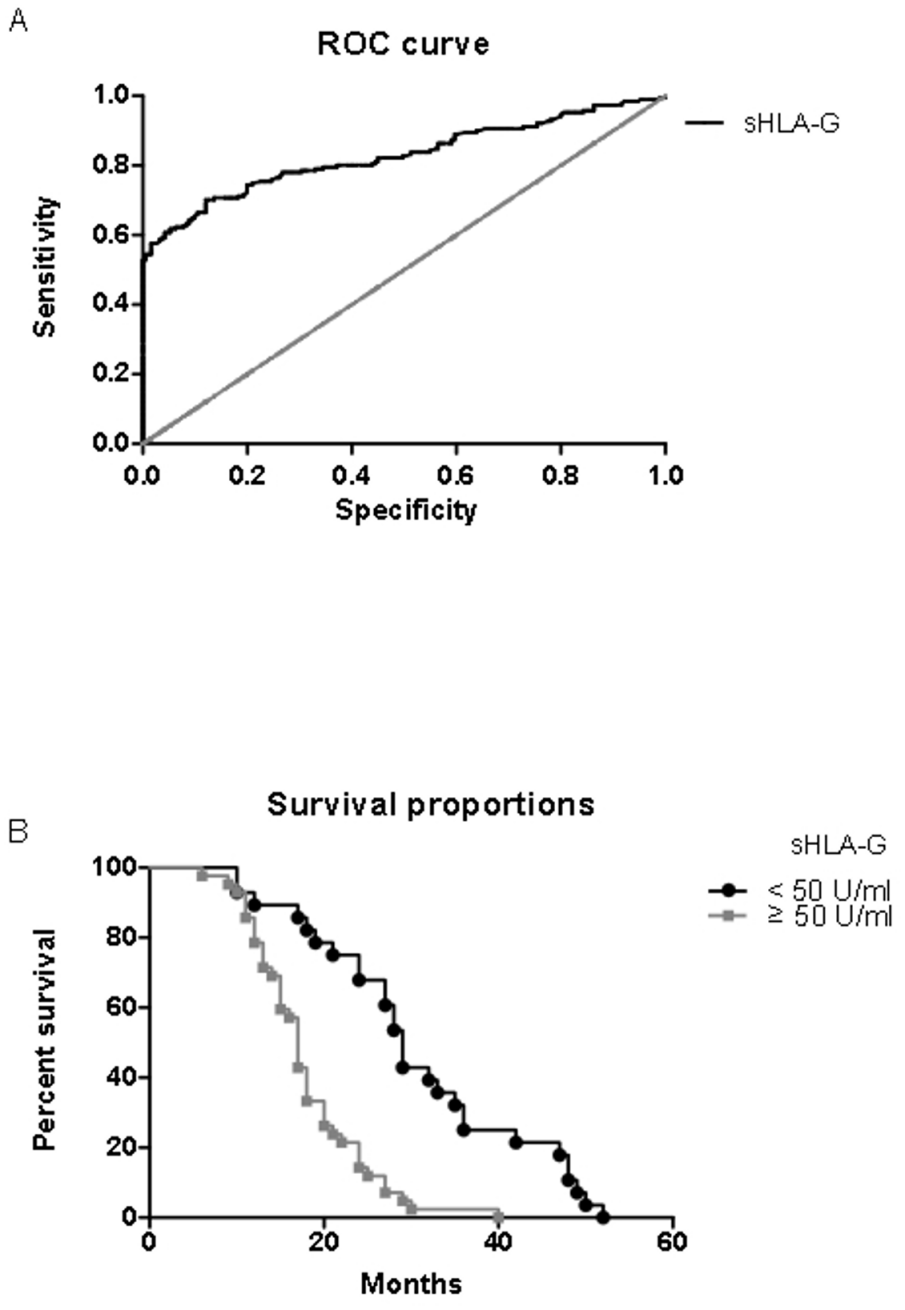
**A. Receiver-operating characteristic (ROC) curve analysis to assess the performance of sHLA-G levels in serum for differentiation between non-small-cell lung carcinoma (NSCLC) patients and healthy controls**. The area under the ROC curve is 0.82 (P< 0.001). B. Kaplan–Meier survival analysis for NSCLC patients. Comparison of the overall survival between the patients with serum sHLA-G levels above and under the median (50 U/ml). (P< 0.0001).

The area under the curves was 0.82 for controls vs patients (Fig 2A). Given 100% specificity, the highest sensitivity achieved to detect NSCLC was 52.8%, at a cutoff value of 24.9 U/ml. (P< 0.001, CI = 0.78–0.87). A total of 135 patients were available for a follow-up study. The follow up period was 50 months or until death. The median average follow-up for surviving patients was 23 months (range, 6–52 months), and during the entire period, there were 70 cancer-related deaths (51.9%). Patients with the sHLA-G above median level (≥ 50 U/ml) had a statistically significant shorter survival time than those with lower sHLA-G expression (P< 0.0001) (Fig 2B).

## Discussion

The most common immune-escape mechanism in experimental or spontaneous tumors is the downregulation or lack of expression of classical HLA class I molecules (HLA-A and -B), which directly affects cytotoxic T cell function against tumor cells [7]. In the absence of classical HLA class I molecules, NK cells become the major actors for tumor elimination. The microenvironment of the tumor or the transformed cells per se may induce HLA-G expression, impairing the activity of NK cells [21]. In addition, HLA-G molecules can be transferred by trogocytosis to competent cytotoxic cells rendering them unresponsive to tumor antigens [22].

Previous studies have demonstrated HLA-G expression in lung cancer tissue [9–11] and relatively high blood sHLA-G levels in patients with NSCLC [12–14]. Two recent studies in Polish patients have found no significant correlations between NSCLC and four polymorphisms in the promoter and/or 3’untranslated region (3’UTR) of *HLA-G* gene [23,24]. However, to date no study has looked at the HLA-G allelic variants. Herein, we report that both *HLA-G* alleles and serum sHLA-G levels are significantly associated with altered risks of NSCLC and clinical disease stages in Tunisians.

The HLA-G*01:*01*:*01* and G*01:*04*:*01* alleles increased risks, whereas the HLA-G*01:*05N* and G*01:*06* alleles conferred protection against both NSCLC and advanced disease stages. Moreover, in agreement with previous studies [12–14], serum sHLA-G levels were higher in patients with NSCLC and in particular those with advanced disease stages compared to healthy subjects. Interestingly, it has been previously shown that individuals carrying the HLA-G*01:*04*:*01* allele have increased blood sHLA-G levels, whereas those harboring the HLA-G*0105N allele, which does not encode a functional HLA-G1 protein, have reduced blood sHLA-G levels [25]. This finding suggests that in the in context of NSCLC, expression of *HLA-G* is under the control of the genetic background of the patients.

The ROC curve analyses were performed to evaluate the feasibility of sHLA-G as a diagnostic marker for NSCLC. Similarly to the study of Cao *et all* [13] this analysis suggests that the measurement of serum sHLA-G levels is a highly specific and sensitive method to identify NSCLC patients. Moreover, serum sHLA-G concentration above the median level (50 U/ml) in Tunisian NSCLC patients is associated with a shorter survival time. Similar findings have been reported in Chinese and German NSCLC patients with plasma sHLA-G levels above 32 U/ml and 40 U/ml, respectively [12–14].

In conclusion, this study demonstrates that *HLA-G* allelic variants are an independent risk factor for NSCLC in the Tunisian population. Serum sHLA-G levels in NSCLC patients could be useful biomarkers for the diagnostic and prognosis of NSCLC.

## Supporting Information

S1 Table Distribution of selected HLA-G genotypes by disease status and histological types. N: number, NSCC: none small cell carcinoma. Selected HLA-G genotypes are those with a frequency above 5% in the study population.
(DOCX)

S2 Table Adjusted OR (CI) for the associations between selected HLA-G genotypes and histological types. CI, confidence interval; N.A: none applicable; NSCC: none small cell carcinoma OR, Odds ratio. Selected HLA-G genotypes are those with a frequency above 5% in the study population. Adjusted ORs for smoking status
(DOCX)

S1 Fig Serum sHLA-G levels in healthy controls and non-small-cell lung carcinoma (NSCLC) patients with different histopathological types. sHLA-G in NSCLC patients with all histopathological types were significantly increased when compared to controls as determined by the Mann-Whitney U test (*** P< 0.0001).
(DOCX)
