# Supplementary material for: Human Leukocyte Antigen G Polymorphism and Expression Are Associated with an Increased Risk of Non-Small-Cell Lung Cancer and Advanced Disease Stage
Source: PLoS One. 2016 Aug 12;11(8):e0161210. doi: 10.1371/journal.pone.0161210 (PMC4982692; doi:10.1371/journal.pone.0161210)
Supplement: S1 Table — N: number, NSCC: none small cell carcinoma. Selected HLA-G genotypes are those with a frequency above 5% in the study population. (DOCX) [file pone.0161210.s001.docx]

**Table S1**

| Genotypes  N | Controls  191 (%) | NSCC  191 (%) | Adenocarcinoma  106 (%) | [Squamous-cell carcinoma](http://en.wikipedia.org/wiki/Squamous-cell_lung_carcinoma)  57 (%) | | | [Large-cell carcinoma](http://en.wikipedia.org/wiki/Large-cell_lung_carcinoma)  28 (%) |
| --- | --- | --- | --- | --- | --- | --- | --- |
| 010101  Homozygote  Heterozygote  Absent | 26 (13.6)  75 (39.3)  90 (47.1) | 17 ( 8.9)  110 (57.6)  64 (33.5) | 4 ( 3.8)  63 (59.4)  39 (36.8) | 9 (15.8)  31 (54.4)  17 (29.8) | | 4 (14.3)  16 (57.1)  8 (28.6) | |
| 010102  Homozygote  Heterozygote  Absent | 5 ( 2.6)  71 (37.2)  115 (60.2) | 7 ( 3.7)  46 (24.0)  138 (72.3) | 3 ( 2.8)  26 (24.5)  77 (72.7) | 2 ( 3.5)  13 (22.8)  42 (73.7) | 2 ( 7.0)  7 (25.0)  19 (68.0) | | |
| 010301  Homozygote  Heterozygote  Absent | 5 ( 2.6)  34 (17.8)  152 (79.6) | 1 ( 0.5)  44 (23.1)  146 (76.4) | 0 ( 0.0)  30 (28.3)  76 (71.7) | 0 ( 0.0)  7 (12.3)  50 (87.7) | 1 ( 3.6)  7 (25.0)  20 (71.4) | | |
| 010401  Homozygote  Heterozygote  Absent | 0 ( 0.0)  15 ( 7.9)  176 (92.1) | 1 ( 0.5)  31 (16.2)  159 (83.3) | 1 (0.94)  17 (16.03)  88 (83.01) | 0 (0.0)  10 (17.54)  47 (82.45) | 0 (0.0)  4 (14.28)  24 (85.71) | | |
| 010404  Homozygote  Heterozygote  Absent | 6 ( 3.1)  29 (15.2)  156 (81.7) | 0 ( 0.0)  24 (12.7)  167 (87.3) | 0 ( 0.0)  14 (13.2)  92 (86.8) | 0 ( 0.0)  7 (12.3)  50 (87.7) | 0 ( 0.0)  2 ( 7.1)  26 (92.9) | | |
| 0105N  Homozygote  Heterozygote  Absent | 0 ( 0.0)  21 (12.0)  170 (98.0) | 0 ( 0.0)  12 ( 6.3)  179 (93.7) | 0 ( 0.0)  6 ( 5.7)  100 (94.3) | 0 ( 0.0)  5 ( 8.8)  52 (91.2) | 0 ( 0.0)  1 ( 3.8)  27 (96.4) | | |
| 0106  Homozygote  Heterozygote  Absent | 0 ( 0.0)  36 (18.8)  155 (81.2) | 14 ( 7.1)  22 (11.7)  155 (81.2) | 10 ( 9.4)  14 (13.2)  82 (77.4) | 4 ( 7.0)  6 (10.5)  47 (82.5) | 0 ( 0.0)  2 ( 7.1)  26 (92.9) | | |
