# Supplementary material for: Human Leukocyte Antigen G Polymorphism and Expression Are Associated with an Increased Risk of Non-Small-Cell Lung Cancer and Advanced Disease Stage
Source: PLoS One. 2016 Aug 12;11(8):e0161210. doi: 10.1371/journal.pone.0161210 (PMC4982692; doi:10.1371/journal.pone.0161210)
Supplement: S2 Table — CI, confidence interval; N.A: none applicable; NSCC: none small cell carcinoma OR, Odds ratio. Selected HLA-G genotypes are those with a frequency above 5% in the study population. Adjusted ORs for smoking status (DOCX) [file pone.0161210.s002.docx]

**Table S2.**

| Genotypes | NSCC | Adenocarcinoma | [squamous-cell carcinoma](http://en.wikipedia.org/wiki/Squamous-cell_lung_carcinoma) | | [large-cell carcinoma](http://en.wikipedia.org/wiki/Large-cell_lung_carcinoma) |
| --- | --- | --- | --- | --- | --- |
| 010101  Homozygote  Heterozygote  Absent | 0.84 [0.39-1.81]  **1.74 [1.05-2.88]**  1.0 | 0.32 [0.10-1.04]  **1.80 [1.03-3.15]**  1.0 | 1.86 [0.68-5.06]  2.03 [0.96-4.30]  1.0 | 3.85 [0.97-15.2]  2.15 [0.83-5.52]  1.0 | |
| 010102  Homozygote  Heterozygote  Absent | 1.39 [0.35-5.60]  0.61 [0.37-1.02]  1.0 | 1.21 [0.23-6.47]  0.61 [0.33-1.10]  1.0 | 1.96 [0.24-16.0]  0.59 [0.28-1.26]  1.0 | 3.71 [0.50-27.5]  0.69 [0.26-1.79]  1.0 | |
| 010301  Homozygote  Hétérozygote  Absent | n.a  1.65 [0.90-3.0]  1.0 | n.a  1.83 [0.95-3.53]  1.0 | n.a  0.98 [0.40-2.40]  1.0 | 1.13 [0.12-11.5]  1.82 [0.66-5.10]  1.0 | |
| 010401  Homozygote  Heterozygote  Absent | n.a  **2.18 [1.02-4.70]**  1.0 | n.a  **2.44 [1.04-5.77]**  1.0 | n.a  2.26 [0.81-6.34]  1.0 | n.a  1.98 [0.55-6.82]  1.0 | |
| 010404  Homozygote  Heterozygote  Absent | n.a  0.92 [0.46-1.88]  1.0 | n.a  0.88 [0.40-1.96]  1.0 | n.a  0.80 [0.30-2.14]  1.0 | n.a  0.53 [0.11-2.52]  1.0 | |
| 0105N  Homozygote  Heterozygote  Absent | n.a  **0.43 [0.19-0.98]**  1.0 | n.a  0.40 [0.15-1.09]  1.0 | n.a  0.64 [0.21-1.93]  1.0 | n.a  0.25 [0.03-2.06]  1.0 | |
| 0106  Homozygote  Heterozygote  Absent | n.a  0.57 [0.29-1.09]  1.0 | n.a  0.70 [0.33-1.46]  1.0 | n.a  0.52 [0.19-1.42]  1.0 | n.a  0.32 [0.07-1.47]  1.0 | |
