# Supplementary figures and images for: Human Leukocyte Antigen G Polymorphism and Expression Are Associated with an Increased Risk of Non-Small-Cell Lung Cancer and Advanced Disease Stage
Source: PLoS One. 2016 Aug 12;11(8):e0161210. doi: 10.1371/journal.pone.0161210 (PMC4982692; doi:10.1371/journal.pone.0161210)

**S1 Figure**


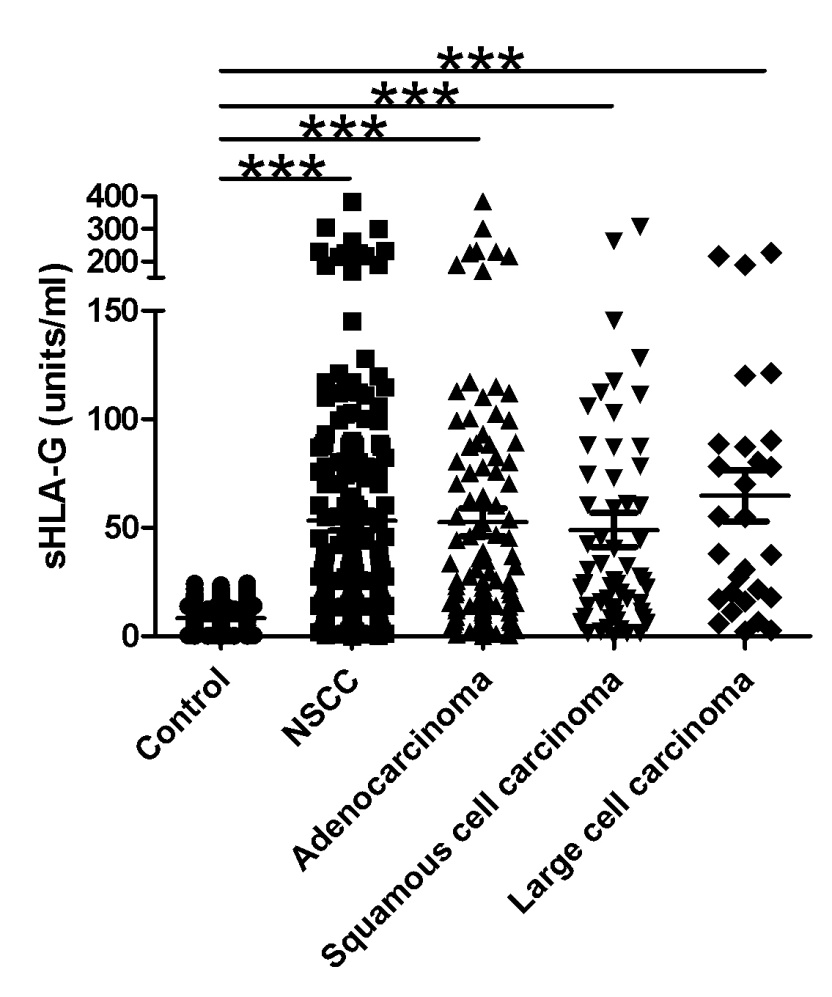

Supplement: S1 Fig — sHLA-G in NSCLC patients with all histopathological types were significantly increased when compared to controls as determined by the Mann-Whitney U test (*** P< 0.0001). (DOCX) [file pone.0161210.s003.docx]
